# Aberrant Periodontal and Systemic Immune Response of Overweight Rodents to Periodontal Infection

**DOI:** 10.1155/2019/9042542

**Published:** 2019-01-03

**Authors:** Ting Yu, Li Zhao, Xin Huang, Baoyi Xie, Jincai Zhang, Dongying Xuan

**Affiliations:** ^1^Department of Periodontology, Key Laboratory of Oral Medicine, Guangzhou Institute of Oral Disease, Stomatology Hospital of Guangzhou Medical University, Guangzhou, China; ^2^Department of Prosthodontics, Guanghua School of Stomatology, Sun Yat-sen University, Guangdong Provincial Key Laboratory of Stomatology, Guangzhou, China; ^3^Department of Periodontology, Guanghua School of Stomatology, Sun Yat-sen University, Guangdong Provincial Key Laboratory of Stomatology, Guangzhou, China; ^4^Laboratory Section, Affiliated Hospital of Stomatology, Southern Medical University, Guangzhou, China; ^5^Savaid Medical School, University of Chinese Academy of Sciences, Beijing, China; ^6^Department of Periodontology, Hangzhou Dental Hospital, Savaid Medical School, University of Chinese Academy of Sciences, Hangzhou, China

## Abstract

This study aimed to explore periodontal and systemic immune response of overweight hosts to periodontitis. Forty C57 BL/6J male mice were divided into high (HF) or low fat (LF) diet groups and fed with the two diets, respectively, for 8 weeks. Each diet group was then divided into periodontitis (P) or control (C) groups (n = 10 per group) for 10-day ligation or sham-ligation. Overweight-related parameters including body weight were measured. Alveolar bone loss (ABL) was morphometrically analyzed and periodontal osteoclasts were stained. Periodontal immune response including leukocyte and macrophage number and inflammatory cytokines were analyzed by histology and quantitative PCR. Serum cytokine and lipid levels were quantified using electrochemiluminescence immunoassays, enzyme-linked immunosorbent assays, and biochemistry. It was found that HF group had 14.4% body weight gain compared with LF group (*P* < 0.01). ABL and periodontal osteoclast, leukocyte, and macrophage number were higher in P group than C group regardless of diet (*P* < 0.05). ABL and periodontal osteoclast number were not affected by diet regardless of ligation or sham-ligation. Leukocyte and macrophage number and protein level of tumor necrosis factor *α* (TNF-*α*) in periodontium and serum interleukin-6 level were downregulated by HF diet in periodontitis mice (*P *< 0.05). Periodontal protein level of TNF-*α* was highly correlated with serum interleukin-6 and low-density lipoprotein cholesterol levels (*P* < 0.01). These findings indicated that impaired immune response occurs both periodontally and systemically in preobesity overweight individuals. Given a well-reported exacerbating effect of obesity on periodontitis, overweight, if let uncontrolled, might place the individuals at potential risk for future periodontal tissue damage.

## 1. Introduction

Obesity and periodontitis, both as chronic inflammatory diseases, share many systemic comorbidities, including cardiovascular diseases and diabetes [1]. Increasing clinical studies indicate the potential link between the two diseases [2], which might put those with systemic comorbidities or periodontal diseases at higher risk. For instance, body mass index (BMI) ≥ 30 was related to more than 3 times of incidence of periodontitis compared with BMI < 20 [3]. However, the linking mechanisms are poorly understood. A most proposed linker is systemic inflammation and impaired immune response, by which obesity might promote aberrant periodontal inflammation and exacerbated alveolar bone loss (ABL) [1, 4, 5]. While many studies focus on the correlation of periodontitis with obesity, few has investigated its correlating mechanism with overweight, a preobesity state which has also been suggested to increase periodontitis risk [6–8]. Given a much higher prevalence of overweight than that of obesity [9], studies on the issue would be more meaningful and provide biological basis for development of early interventions on obesity-related comorbidities including periodontitis.

Diet-induced obesity (DIO) model simulates diet-related obesity of human the best among various obesity models and thus gets widely used in animal studies [10]. Its establishment depends on excessive intake of high-fat (HF) diet over time, generally for about 15 weeks [10]. However, there have been part of immunometabolic dysregulations much early (i.e., 4 to 8 weeks on diet) before a full picture of metabolic syndrome (including obesity) occurs [11–16]. The partially aberrant state at early stages is more like overweight than like obesity, which has also been found to increase the risk of some infection or inflammatory diseases as obesity does [12, 17, 18]. Therefore, it would be of interest to test a relationship between diet-induced overweight (DIOW) and periodontal infection. Previous animal studies have rarely used DIOW model to explore potential linking mechanisms between the two conditions. Instead, they generally used DIO animals and found impaired immune response periodontally or systemically to periodontal infection and showed exacerbating effect of obesity on ABL [19–24]. Hence, the present study aimed to explore periodontal and systemic immune response of overweight hosts to periodontal infection.

## 2. Materials and Methods

### 2.1. DIOW Model

The animals were provided by and cultured in Guangdong Medical Laboratory Animal Center (GMLAC). The animal study was conducted under the approval of Animal Ethics Committee of GMLAC and in accordance with the National Institutes of Health guide for the care and use of laboratory animals [25].

Forty C57 BL/6J mice (male, 6-week old) were randomly divided into high (HF) or low fat (LF) diet groups (n = 20 per group) and fed with 60 kcal% HF diet (Research Diet, New Brunswick, NJ) or 11 kcal% LF laboratory diet, respectively, for 8 weeks. The mice were individually housed* ad libitum* in specific-pathogen-free environment. Body weight was measured once a week. Sixteen-hour (overnight) fasting blood glucose (FBG) was measured by a glucometer (OneTouch Ultra, Johnson & Johnson, Shanghai, China) at week 0 and 8.

### 2.2. Experimental Periodontitis and Tissue Harvest

After 8 weeks on diet, the mice were transferred into conventional environment. Each diet group was divided into periodontitis (P) or control (C) groups (n = 10 per group).* Porphyromonas gingivalis* (*Pg*) ATCC33277 (ATCC, Manassas, VA) was cultured as described [26]. Under anesthesia with 10% chloral hydrate (i.p.), P group was ligated bilaterally at maxillary second molars with* Pg*-adhered silk (Ethicon, Johnson & Johnson, Shanghai, China) for 10 days. For control, C group was anesthetized and sham-ligated with sterile silk, which was removed at once.

At day 10, the mice were euthanized by cardiac puncture. Fasting serum was separated. Visceral adipose tissue at epididymal (eAT), perirenal (pAT), and mesenteric (mAT) sites was weighed. Organ weight percentage was calculated by dividing organ weight by body weight. One side of the upper jaw was fixed in 10% neutral formaldehyde for histology and the other side was for RNA extraction and morphometric analysis.

### 2.3. Morphometric Analysis of ABL

The bone was fleshed and stained with 1% methylene blue (MP Biomedicals, Shanghai, China) as described previously [27]. The frontal view of the bone was captured under a digital microscope system (Leica Microsystems, GmbH, Wetzlar, Germany). Distance from cementoenamel junction to alveolar bone crest (CEJ-ABC) at 18 sites of the three molars was measured and averaged as vertical bone loss [28]. The measurement was repeated independently by three operators.

### 2.4. Leukocyte Counting in Periodontium

The alveolar bone was fixed for 2 days, decalcified in 10% ethylene diamine tetraacetic acid (EDTA) for 2 weeks at room temperature, and then dehydrated and embedded. Five-*μ*m sagittal sections were obtained and stained with Hematoxylin & Eosin (H&E). Infiltrated leukocytes around the second molar were counted (400×) as described in our previous study [29].

### 2.5. Tartrate-Resistant Acid Phosphatase (TRAP) Staining and Osteoclast Counting

Alveolar bone sections were stained with TRAP using a commercial kit (Sigma-Aldrich, St. Louis, MO). Multinucleated TRAP^+^ cells (i.e., osteoclasts) near the bone surface around the second molar were counted (200×) as described previously [29].

### 2.6. Immunohistochemical Analysis of Macrophages and Inflammatory Cytokines in Periodontium

After dewaxing and hydration of the alveolar bone section, antigen retrieval was done in boiling Tris-EDTA solution (pH = 9.0) for 15 to 20 minutes. Endogenous peroxidase was blocked by 3% hydrogen peroxide, followed by incubation with 10% normal goat serum. Incubation with primary antibodies (Abcam, Cambridge, MA) anti-CD68 (1:100, ab31630), tumor necrosis factor-*α* (TNF-*α*) (1:100, ab6671), interleukin (IL)-1*β* (10 *μ*g/mL, ab9722), or IL-10 (1:100, ab9969) was done, respectively, for 2 or 3 hours at 37 centigrade. Incubation with a ready-to-use secondary antibody coupled with horseradish peroxidase (goat-anti-rabbit) (Polink-1, Golden Bridge International, Mukilteo, WA) was done for 30 minutes at 37 centigrade. For color reaction, 3,3′-diaminobenzidine was used. Phosphate buffered saline was used for blank control. The tissue was counterstained with hematoxylin. CD68^+^ cells (i.e., macrophages) around the second molar were counted and calculated the same as leukocyte counting. Protein levels were quantified as integrated optical density/area of interest (i.e., mean optical density) using image analysis software (Image Pro Plus, Media Cybernetics, Silver Spring, MD). Area of interest was restricted to the suprabony soft tissue mesial or distal to the second molar (200×). For each target protein, three discontinuous sections were stained for analysis.

### 2.7. Quantitative PCR Analysis of IL-6 in Gingiva

Gingiva was stripped from the upper jaw. Total RNA was extracted, reversely translated, and semiquantified by fluorescent quantitative PCR (RNAiso Plus/PrimeScript RT reagent kit/SYBR Premix Ex Taq PCR kit, Takara Bio, Otsu, Japan) using a real-time PCR analyzing system (ViiA 7, Applied Biosystems, Waltham, MA). The mRNA level of IL6 was detected, with glyceraldehyde-3-phosphate dehydrogenase (GAPDH) as endogenous control. The relative mRNA levels were calculated by 2^-ΔΔCt^ method. The primers used were referred to in a nonprofit platform (PrimerBank, Harvard University, Cambridge, MA) as follows (5′ to 3′, forward and reverse).* Gapdh*, AGGTCGGTGTGAACGGATTTG and GGGGTCGTTGATGGCAACA;* Il6*, CTGCAAGAGACTTCCATCCAG and AGTGGTATAGACAGGTCTGTTGG.

### 2.8. Serological Analysis of Insulin, Inflammatory Cytokines, and Lipids

Fasting serum insulin (Fins) (ALPCO, Windham, NH) and C-reactive protein (CRP) (R&D system, Bio-Techne, Minneapolis, MN) were detected by two highly sensitive enzyme-linked immunosorbent assay kits. Serum TNF-*α* and IL-1*β*, -6, and -10 were detected by electrochemiluminescence immunoassays (V-Plex, Meso Scale Discovery, Gaithersburg, MD). Serum lipids including triglyceride (TG), total cholesterol (TC), low-density lipoprotein cholesterol (LDLC), and high-density lipoprotein cholesterol (HDLC) were quantified as previously described [30].

### 2.9. Statistics

Statistical software (SPSS 17.0, IBM, Armonk, NY) was used. For factorial design, data were analyzed by 2-way analysis of variance (ANOVA), with diet and ligation as main effects. Simple effect or comparison between two independent samples was analyzed by 1-way ANOVA (Welch adjustment was used with unequal variances). Multiple linear regression models were tested between periodontal TNF-*α* and serum cytokines or lipids. Data are presented as means ± standard deviations. Statistical significance was considered at* P *< 0.05.

**Figure 1:**
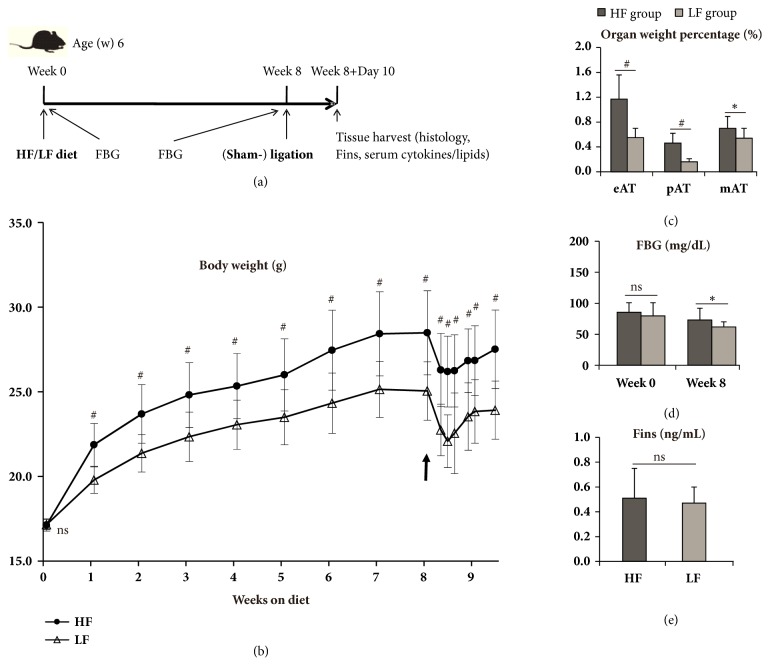
Timelines of the animal study (a) and DIOW-related parameters (b-e). Body weight was monitored at day 0, 2, 3, 4, 6, 7, and 10 during the 10-day ligation. The arrow (b) indicates time point for experimental periodontitis. ns, no significance; *∗*,* P* < 0.05; #,* P* < 0.01. n = 14 to 20 per group.

The timeline for animal treatment is shown in Figure 1(a).

## 3. Results

### 3.1. DIOW Model

After 8-week diet, HF group demonstrated 14.4% body weight gain compared with that of LF group (29.4 vs. 25.7 (g),* P* < 0.01) (Figure 1(b)). After 10-day ligation/sham-ligation, HF group still exhibited 15.5% weight gain (28.3 vs. 24.5 (g),* P* < 0.01) and showed enhanced weight percentage of all types of visceral adipose tissue (*P* < 0.05) and FBG level (73.2 vs. 61.8 (mg/dL), P < 0.05) but comparable Fins level relative to those of LF group (Figures 1(c)–1(e)).

### 3.2. ABL and Osteoclast Amount

**Figure 2:**
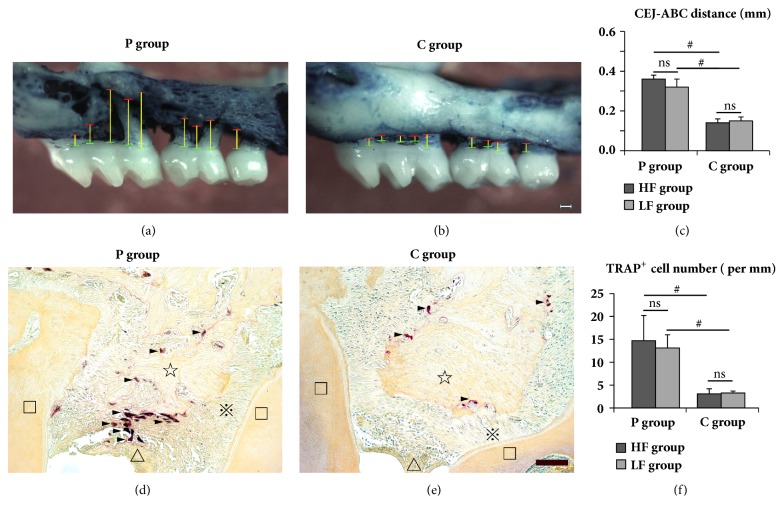
ABL (a-c) and osteoclast amount (d-f) in different diet and ligation groups. Alveolar bone and periodontal sections were stained with methylene blue (a and b, 30×) and TRAP (d and e, 200×), respectively. Green/red/yellow lines: CEJ level, ABC level, and distance from CEJ to ABC, respectively. Black/hollow triangles: TRAP^+^ cells and gingival epithelium, respectively. □/☆/*※*: dentine, alveolar bone, and periodontal ligament, respectively. ns, no significance; #,* P* < 0.01. n = 6 to 8 per group. White/black scale bars: 150 and 100 *μ*m, respectively.

Within both diet groups, P group had more ABL and osteoclast (TRAP^+^) number in periodontium than those of C group (*P* < 0.05). However, ABL and osteoclast number were not affected by diet regardless of ligation or sham-ligation (Figure 2).

### 3.3. Periodontal Leukocyte and Macrophage Amount and Cytokine Levels

**Figure 3:**
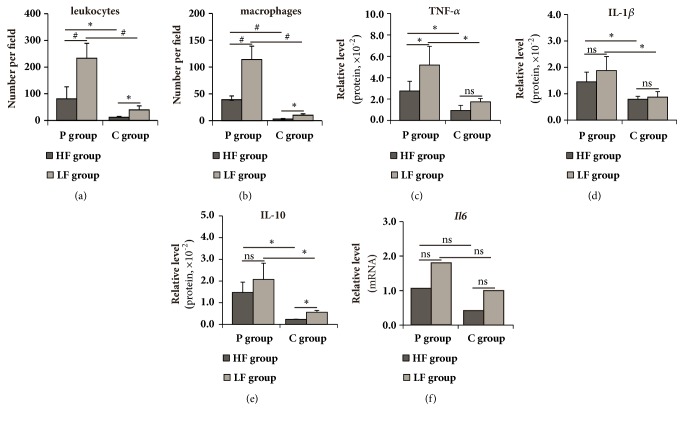
Periodontal leukocyte and macrophage amount and cytokine levels in different diet and ligation groups. ns, no significance; *∗*,* P* < 0.05; #,* P* < 0.01. n = 3 to 5 per group.

Regardless of diet, P group showed more leukocyte and macrophage (CD68^+^) number in periodontium compared with those of C group (*P* < 0.05) (Figures 3(a) and 3(b)). It was the same case for protein levels of pro- (TNF-*α* and IL-1*β*) and anti-inflammatory cytokines (IL-10) in periodontium (*P* < 0.05) (Figures 3(c)–3(e)). Ligation had significant main effect (*F* = 7.23,* P* < 0.05) but no significant simple effect on the mRNA level of IL-6 (Figure 3(f)), which was doubled in P group relative to that of C group, as shown by 2-way ANOVA.

Regardless of ligation or sham-ligation, HF group exhibited reduced leukocyte and macrophage number compared to those of LF group (*P* < 0.05). Within P group, HF group showed reduced protein level of TNF-*α* (HFP vs. LFP,* P* < 0.05) and a tendency of downregulated protein levels of IL-1*β* and IL-10 relative to those of LF group. It was the same case for protein level of IL-10 within C group (HFC vs. LFC,* P* < 0.05). Diet had no significant main effect on mRNA level of IL-6 (*F* = 1.58,* P* > 0.05).

**Figure 4:**
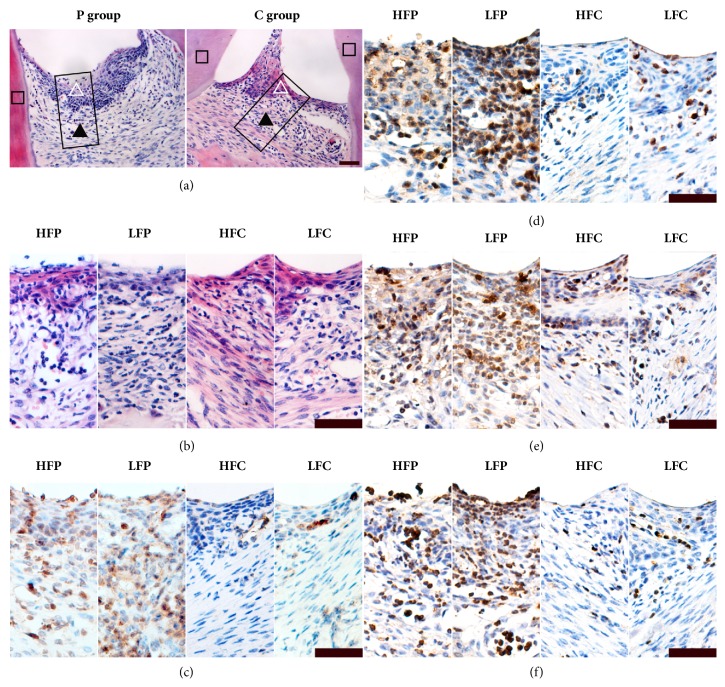
Illustrations of periodontal leukocytes and macrophage and cytokine immunostaining. Rectangles in the H&E-stained sections (a) indicate a representative area from which the partial enlarged views of the immunostained sections (b-f) originate. Hollow/black triangles and square indicate gingival epithelium, subepithelial connective tissue, and dentine, respectively (a). Leukocytes were counted in H&E-stained sections (b). Macrophage (c) and TNF-*α* (d), IL-1*β* (e), and IL-10 (f) were stained in brown (400×). Scale bars, 50 *μ*m.

Figure 4 demonstrated representative images for leukocytes, macrophages, and cytokines in periodontal sections in different diet and ligation groups.

### 3.4. Serum Cytokine Levels

**Figure 5:**
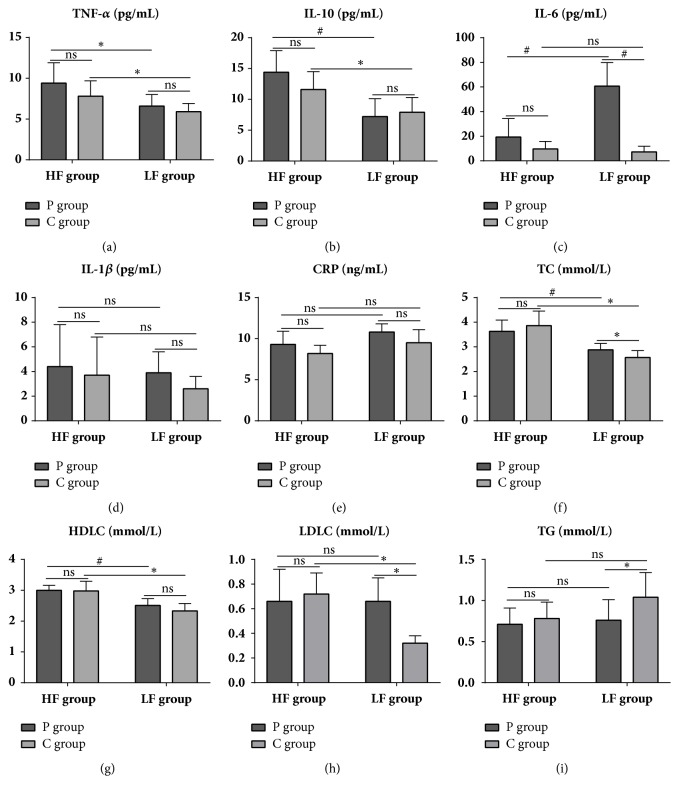
Serum cytokine (a-e) and lipid (f-i) levels in different diet and ligation groups. ns, no significance; *∗*,* P* < 0.05; #,* P* < 0.01. Cytokines, n = 4 to 8 per group; lipids, n = 7 to 10 per group.

Whether with ligation or not, HF group displayed enhanced TNF-*α* and IL-10 levels in serum relative to those of LF group (*P* < 0.05) (Figures 5(a) and 5(b)). Within LF group, IL-6 level was upregulated in P group compared with that of C group (LFP vs. LFC,* P* < 0.01), whereas, within P group, IL-6 level was downregulated in HF group compared to that of LF group (HFP vs. LFP,* P* < 0.01) (Figure 5(c)). IL-1*β* and CRP levels were unaffected by either diet or ligation (Figures 5(d) and 5(e)).

### 3.5. Serum Lipid Levels

Regardless of ligation or not, HF group demonstrated higher TC and HDLC levels in serum compared with those of LF group (*P* < 0.01) (Figures 5(f) and 5(g)). Within C group, HF group exhibited elevated LDLC level compared to that of LF group (HFC vs. LFC,* P* < 0.01) (Figure 5(h)).

Within LF group, P group displayed increased TC and LDLC levels (LFP vs. LFC,* P* < 0.05) and a tendency of upregulated HDLC level but decreased TG level (*P* < 0.05) compared to those of C group (Figures 5(f)–5(i)). Within HF group, however, none of the lipids were affected by ligation.

### 3.6. Multiple Linear Regression

Table 1 showed the final multiple linear regression models for periodontal TNF-*α* level alongside independent variables regarding serum cytokine or lipid levels. It revealed that periodontal TNF-*α* level was positively related to serum IL-6 (*β* = 0.060,* P *< 0.01) and LDLC (*β* = 7.220,* P *< 0.01) levels, which accounted for 98% and 73% (*R*^2^), respectively, of the former's variations.

## 4. Discussion

The present study explored the linking mechanism between overweight and periodontitis from the viewpoint of impaired immune response. Despite the absence of exacerbated ABL or osteoclastogenesis by overweight, the condition reduced periodontal macrophage number and partially suppressed pro- (i.e., TNF-*α* and IL-6) and anti-inflammatory cytokines (IL-10) in periodontium and circulation in periodontitis mice. The change of periodontal TNF-*α* level was well predicted by serum IL-6 and LDLC levels. These findings revealed an impaired systemic and periodontal immune response of overweight individuals to periodontitis.

According to human standards, 25% < BMI < 29% and BMI > 30% correspond to overweight and obesity, respectively [31]. BMI > 30% is approximately equal to > 25% body weight gain [32]. Despite no definite standard on obesity or overweight for laboratory animals, > 10 g or > 20%–25% body weight gain is generally considered obesity standard for animals, which needs about 15-week feeding on HF diet [10]. At the stage, animals show full pictures of obesity including hyper-glycemia, -lipidemia, and -insulinemia and central obesity [33]. However, many studies have observed early development of impaired immune response in rodents fed for only 4 to 8 weeks, such as adipose tissue inflammation [11] and enhanced response of some serum cytokines [12–16]. It indicates that ≤ 8-week HF diet feeding induces an aberrant state of preobesity overweight. In our study, 8-week HF feeding to mice induced mild body weight gain (14.4% or 3.7 g), increased FBG level within the normal range (< 126 mg/dL [34]), and upregulated levels of a part of serum cytokines (TNF-*α* and IL-10) and lipids (TC and HDLC) without hyperinsulinemia. The findings accorded with a feature of overweight model.

With the DIOW model, we found a partially suppressed periodontal and serum cytokine response of DIOW mice to periodontitis, consistent with our previous study and some reports which used DIO animals with a similar infection duration [19, 20]. However, studies regarding DIO have also demonstrated a differentiated response of proinflammatory cytokines to periodontitis in periodontium or circulation in obese animals [19–24]. Some studies showed solely downregulated [19, 20] or upregulated response [22, 24] while others exhibited bidirectionally changed one [21, 23] in proinflammatory cytokines. The differentiated cytokine response seems associated more with infection stage than with induction method (ligation, oral gavage, or i.v. injection) or pathogen type (*Actinobacillus actinomycetemcomitans*,* Pg* or their lipopolysaccharide (LPS), or* Escherichia coli LPS*). For instance, infections for < 20 days tend to induce partially hypoactivated cytokine response [19–21, 35], while those for > 4 weeks tend to cause hyperactivated one [22, 24]. This phenomenon might indicate a dysregulated host-parasite interaction modified by overweight/obesity in both early and later stages of infection. In early stages, as proposed, partially suppressed inflammatory response might be helpful for bacteria's evasion and colonization [36]. In later stages, hyperactivated unresolved inflammation could provide adequate nutrients to established biofilm and cause self tissue damage [36, 37].

DIOW seems to impair cytokine response less extensive than DIO does, given that DIO was also found to suppress TNF-*α*, CRP, and IL-10 levels in serum and reduce IL-1*β* level in periodontium while the current study did not [19, 23]. Suppressed inflammatory response would cause reduced phagocytosis, antigen uptake, and production of microbicidal molecules in host immune cells, which would cause direct tissue damage, including degradation of extracellular matrix and ABL, by bacterial virulence factors [38, 39]. Unlike some studies which found enhanced ABL or osteoclastogenesis by DIO [19–24, 40], we did not observe the same consequence in DIOW mice, possibly due to the relatively slight impairment of immune response. However, the consequence might logically happen if overweight was let uncontrolled. In this context, interventions on overweight-related impaired immune response might be more meaningful for prevention of their comorbidities including periodontitis.

Biological mechanisms regarding the partially suppressed immune response are poorly understood. Some studies have related it to dysregulated macrophage function [19–21, 23, 41]. In this study, we found reduced macrophage number by DIOW in periodontitis mice, suggesting decreased recruitment of monocytes to infection focus because we also found downregulated monocyte chemoattractant protein-1 level by DIO in our previous study [19]. TNF-*α*, IL-1*β*, and IL-10 are early-response mediators, which are largely secreted by innate immune cells including macrophages [42, 43]. Thus, less macrophage infiltration might mean less expression of inflammatory cytokines, as supported by our results that macrophage number was highly related to leukocyte number (*R* = 0.924) and levels of TNF-*α* (*R* = 0.752), IL-1*β* (*R* = 0.775), and IL-10 (*R* = 0.807) in periodontium (*P* < 0.01). Amar et al. have suggested immune tolerance to explain the blunted immune response to periodontal infection in obese hosts [5]. Namely, macrophages in obese individuals are tolerized by chronic and low-level inflammatory stimuli such as free fatty acid, which blunts their inflammatory response to further infection of* Pg* or its components [5, 21, 44]. Interestingly, the blunted macrophage response seems related to biased arginine catabolism, which controls macrophage M1/M2 phenotypes, as manifested by enhanced arginase/inducible nitric oxide synthase ratio and inhibited M1 polarization [29, 41]. Reversely, adoptive transfer of M1 macrophages has been found to improve ABL in periodontitis mice during 5-day infection and inhibition of arginase could prevent the blunted inflammatory response to periodontal pathogens in DIO-educated macrophages [41, 45]. Taken together, dysregulated macrophage phenotypes might be implicated in the pathogenesis of overweight/obesity related periodontal destruction. Functional analysis on macrophage subsets would be needed to reveal respective roles before identifying responsible targets for bone resorption or repair.

An interesting finding in this study was that LDLC level was enhanced by either periodontitis or overweight alone, but unaffected by the two factors together. Moreover, serum LDLC level well predicted periodontal TNF-*α* level, suggesting a playing role of LDLC in the partially suppressed cytokine response to periodontitis in overweight individuals. As proposed, hyperlipidemia could protect hosts from harmful effects of infection in early stages [46]. For instance, lipoproteins could bind and inactivate LPS to lower the latter's proinflammatory effects, which is mediated by apolipoprotein E [47]. In this context, the slight hyperlipidemia in DIOW mice might be adequate to buffer periodontitis-induced endotoxemia without further elevation of LDLC. However, it was unknown why only LDLC rather than other serum lipids participated in the association with the blunted cytokine response in the context of overweight and periodontitis. This priority might be related to LDLC receptor, a mediator dominating both the internalization of lipoprotein-LPS complex and removal of LDLC in circulation [48].

The current study had some limitations. First, 10 days' ligation seemed to induce an acute form of periodontal destruction in mice, which might have limitation to reflect the chronic nature of periodontitis in human. Dynamic evaluations on inflammatory changes at both early and later stages of infection in overweight/obese individuals should be conducted in future studies. Second, additional DIO controls would be expected to show obesity's effects on periodontitis and its difference from overweight's. However, our recent studies have indicated a poor comparability between the two models in the context of periodontal ligation or sham-ligation because of an unneglectable imbalance of postoperative weight loss (POWL) between them [30, 49]. It was found that the POWL in DIO model doubled that in DIOW one (8.6% vs. 4.0%), which could be a significant factor to confound the true effects of obesity or its severity on immunometabolic parameters [30, 49]. POWL seems to be inevitable in ligation-induced periodontitis model mainly due to anesthetic adverse effects especially when it goes with DIO models [30]. In this context, experimental periodontitis models free of anesthesia, such as oral gavage model, could be an alternative to go with DIO(W) models, which might be better for distinguishing the obesity's effects on periodontitis from overweight's. Third, the inflammatory cytokines we detected in periodontium are also secreted by some cell types (e.g., fibroblasts and osteoblasts) other than macrophages [43]. For instance, adaptive immune cells (e.g., B cell) and gingival fibroblasts have been also implicated in obesity-related impairment of immune response to periodontal infection [50, 51]. A broader screening on immunometabolic alterations at molecular and cell levels would be necessary henceforth. Finally, unlike some study that found overactivated periodontal immune response in DIO rodent without any stimulation (including sham operation) [52], we found partially suppressed periodontal immune response (i.e., reduced leukocytes including macrophages and IL-10 level) even in the sham-ligated DIOW mice versus normal weight control. This phenomenon might point to some confounding effect of ligation-related trauma-induced gingival inflammation [28]. However, the confounding effect, if any, relative to infection itself was likely weak [28], considering that leukocyte number in periodontitis mice was 6 to 7 times of that of sham-ligation control.

## 5. Conclusions

This animal study offered new evidence to support the conclusion that impaired immune response occurs both periodontally and systemically in preobesity overweight individuals. Given a well-reported exacerbating effect of obesity on periodontitis, overweight, if let uncontrolled, might place the individuals at potential risk for future periodontal tissue damage.

## Figures and Tables

**Table 1:** Multiple linear regression analysis between periodontal TNF-*α* level and serum cytokine or lipid levels.

Models	Independent variable	Dependent variable periodontal TNF-*α* level			
		Coef.	*t*-value	*P*-value	*R* ^2^
1	Constant	1.076	5.658	0.011	
	Serum IL-6 (included)	0.060	13.633	<0.001	0.984
	Serum TNF-*α* (excluded)	-0.008	-0.084	0.941	
	Serum IL-10 (excluded)	0.113	1.941	0.192	
	Serum IL-1*β* (excluded)	-0.613	-1.518	0.268	
	Serum CRP (excluded)	-0.075	-0.992	0.426	
2	Constant	-0.379	-0.507	0.622	
	Serum LDLC (included)	7.220	5.447	<0.001	0.730
	Serum TG (excluded)	0.135	0.851	0.415	
	Serum TC (excluded)	-0.242	-1.261	0.236	
	Serum HDLC (excluded)	-0.249	-1.633	0.134	

## Data Availability

The data used to support the findings of this study are available from the corresponding author upon request.
